# High prevalence of herpes simplex virus (HSV)- type 2 co-infection among HIV-positive women in Ukraine, but no increased HIV mother-to-child transmission risk

**DOI:** 10.1186/s12884-016-0887-y

**Published:** 2016-04-27

**Authors:** Karoline Aebi-Popp, Heather Bailey, Ruslan Malyuta, Alla Volokha, Claire Thorne, The Ukraine European Collaborative Study in EuroCoord

**Affiliations:** Division of Infectious Diseases, University Hospital Bern, CH-3010 Bern, Switzerland; Population, Policy and Practice Programme, UCL Institute of Child Health, University College London, London, UK; Perinatal Prevention of AIDS Initiative, Odessa, Ukraine; Shupyk National Medical Academy of Postgraduate Education, Kiev, Ukraine

**Keywords:** HIV, Pregnancy, Herpes simplex virus, Mother to child transmission

## Abstract

**Background:**

Over 3500 HIV-positive women give birth annually in Ukraine, a setting with high prevalence of sexually transmitted infections. Herpes simplex virus Type 2 (HSV-2) co-infection may increase HIV mother-to-child transmission (MTCT) risk. We explored factors associated with HSV-2 seropositivity among HIV-positive women in Ukraine, and its impact on HIV MTCT.

**Methods:**

Data on 1513 HIV-positive women enrolled in the Ukraine European Collaborative Study from 2007 to 2012 were analysed. Poisson and logistic regression models respectively were fit to investigate factors associated with HSV-2 seropositivity and HIV MTCT.

**Results:**

Median maternal age was 27 years (IQR 24–31), 53 % (796/1513) had been diagnosed with HIV during their most recent pregnancy and 20 % had a history of injecting drugs. Median antenatal CD4 count was 430 cells/mm^3^ (IQR 290–580). Ninety-six percent had received antiretroviral therapy antenatally. HSV-2 seroprevalence was 68 % (1026/1513). In adjusted analyses, factors associated with HSV-2 antibodies were history of pregnancy termination (APR 1.30 (95 % CI 1.18–1.43) for ≥2 vs. 0), having an HIV-positive partner (APR 1.15 (95 % CI 1.05–1.26) vs partner’s HIV status unknown) and HCV seropositivity (APR 1.23 (95 % CI 1.13–1.35)). The overall HIV MTCT rate was 2.80 % (95 % CI 1.98–3.84); no increased HIV MTCT risk was detected among HSV-2 seropositive women after adjusting for known risk factors (AOR 1.43 (95 % CI 0.54–3.77).

**Conclusion:**

No increased risk of HIV MTCT was detected among the 68 % of HIV-positive women with antibodies to HSV-2, in this population with an overall HIV MTCT rate of 2.8 %. Markers of ongoing sexual risk among HIV-positive HSV-2 seronegative women indicate the importance of interventions to prevent primary HSV-2 infection during pregnancy in this high-risk group.

## Background

In Ukraine HIV prevalence among pregnant women was 0.8–1.0 % between 2009 and 2013, and over 3500 HIV-positive pregnant women gave birth in 2013 [1]. Initially injecting drug use drove the HIV epidemic in Eastern Europe [2] but the main mode of acquisition shifted to heterosexual transmission in 2008 [1] and 45 % of new HIV infections in 2013 were in women. After the collapse of the Soviet Union, social changes and poor public health provision resulted in an epidemic of sexually transmitted infections (STIs), particularly syphilis. Although syphilis rates subsequently declined, rates of other STIs remain higher in Eastern than Western Europe [3]; in a study of pregnant HIV-positive women delivering in 1999–2005, those in Ukraine had a 10-fold higher probability of having syphilis, chlamydia or *Trichomonas vaginalis* than their counterparts in Western Europe [4].

Prevalence of Herpes simplex virus Type 2 (HSV-2) infection, the leading cause of genital ulcer disease worldwide, is around 17–22 % in adults across Europe and the United States with higher prevalence in HIV-positive individuals (35–40 %) [5]. It is well established that genital ulcer disease caused by HSV-2 increases risk of HIV sexual acquisition [6]. During latency HSV-2 is able to avoid clearance by the immune system, but laboratory tests show specific antibodies and it can cause recurrent activations, which are more common and of longer duration with HIV co-infection [7, 8]. This synergistic relationship between HIV and HSV-2 also results in more frequent subclinical episodes of HSV-2 reactivation in HIV-positive individuals, which seems to be associated with increases in plasma and genital tract HIV viral load [9].

Mother-to-child-transmission (MTCT) rates of HIV around 0.5 % have now been reported from Western Europe [10, 11]. In Ukraine, the prevention of MTCT (PMTCT) programme has achieved considerable success, with MTCT rates reduced from 15 % in 2001 to 7 % in 2007 and around 4 % in 2010, following introduction of WHO Option B from 2007 [12, 13] (i.e. combined antiretroviral therapy for all HIV-positive pregnant women). Recent national reporting from Ukraine indicated a MTCT rate of 4.3 % in 2012 [1]. Some data suggest an increased risk of HIV MTCT for HSV-2/HIV co-infected women in general [14] or with genital ulcers [15] and a several trials showed decreased HIV RNA levels in plasma or breastmilk after treatment with Valacyclovir [16, 17].

Our primary aim was to investigate the influence of HSV-2 co-infection on HIV MTCT risk in Ukraine. Secondary aims were to describe the prevalence of HSV-2 antibodies among HIV-infected childbearing women and associated risk factors.

## Methods

### Study setting and subjects

The European Collaborative Study (ECS) is a consented cohort study of HIV-positive pregnant women and their infants, which has enrolled women at regional HIV/AIDS centres in Ukraine since 2000. Women diagnosed with HIV before or during pregnancy or intrapartum and delivering a live-born infant are eligible. Linked anonymous data on maternal, delivery and infant characteristics are collected on standard questionnaires [13]. Between 2007 and 2012, HIV-positive women were enrolled at five centres (Odessa, Kyiv, Donetsk, Mykolaiv and Krivoy Rog) into a nested postnatal sub-study, normally within 3–6 months of delivery, with the aim of obtaining longitudinal information following delivery. Postnatal women self-reported information on socio-demographic characteristics and health behaviours (including drug use, access to contraception and HIV status of current partner) while clinicians provided clinical information [18]. Ethical approval has been obtained from the Great Ormond Street Hospital for Children NHS Trust/Institute of Child Health Research Ethics Committee (reference 96EB02). We obtained also local approvals from all the participating HIV/AIDS Centres.

The study population for this analysis was 1513 women and their infants enrolled in the Ukraine ECS since 2007, with linked data from the postnatal cohort on maternal HSV-2 serostatus.

### Definitions

Injecting drug use (IDU) history was defined as self-report, clinician’s assessment, or neonatal abstinence syndrome in the infant. We used a threshold of HIV RNA <75 copies/ml to define a viral load (VL) as undetectable.

Infants with at least one positive virological marker of infection at any age and/or persistence of antibodies beyond 18 months of age were considered to be HIV-positive, while infants with a negative PCR test result for HIV DNA or RNA and/or negative HIV antibody test were classified as HIV-negative, regardless of age (excluding negative PCR test results on the day of delivery). Infants with conflicting results or without data available were categorised as having an indeterminate HIV status. For multiple births, data on MTCT were based on the first-born infant only unless infection status was discordant between twins/triplets, in which case the infected infant was retained.

Combined antiretroviral therapy (cART) was defined as ≥3 antiretroviral drugs taken simultaneously; 12 women receiving antenatal dual therapy were included in the monotherapy group throughout. Elective caesarean section was defined as before rupture of membranes and onset of labour.

### Data analysis

Univariable comparisons were assessed with the chi-square test or Fisher’s exact test for categorical variables and the Wilcoxon rank sum test for continuous variables. Poisson regression models with robust variance estimators were fitted to obtain prevalence ratios (PR) and adjusted prevalence ratios (aPR) and 95 % confidence intervals (CI) in analyses investigating factors associated with positive HSV-2 serostatus. Socio-demographic characteristics and co-infections associated with HSV-2 seropositivity in univariable analyses (*p* <0.1) were included in the multivariable model, with the exception of those which were considered likely to have post-dated HSV-2 seroconversion.

Logistic regression models fitted to investigate the impact of HSV-2 seropositivity on risk of HIV MTCT were adjusted a priori for factors previously found to be associated with HIV MTCT in this cohort (use of antenatal ART, mode of delivery, preterm delivery and IDU history) [13] and time period of delivery (2007–09 vs 2010–12) and centre, which was included as a random effect to account for regional differences in clinical practice and in maternal characteristics. Sub-analyses were conducted to explore the association between HSV-2 seropositivity and HIV MTCT among (a) mother-child pairs with maternal syphilis serology, as positive syphilis serology has previously been associated with HIV MTCT risk in this population [19]; (b) women who delivered vaginally; (c) women who did not receive antenatal cART.

Statistical analysis was performed using STATA version 12 (Stata Corp LP, College Station USA).

## Results

The 1513 HIV-positive women with HSV-2 serostatus available were enrolled into the postnatal cohort a median of 3.9 months (interquartile range (IQR) 1.1–10.3) after delivery. Median maternal age was 27.3 years (IQR 24.1–30.8) and 87 % (1306/1509) were married or cohabiting. Just under half (647/1423) were primiparous, and 53 % (796/1513) had been diagnosed as HIV-infected during their most recent pregnancy, with 143 (9 %) diagnosed in the third trimester or during delivery. A fifth (295/1504) of women had an IDU history and a similar proportion (286/1464) had a sexual partner who injected drugs.

Median antenatal CD4 count measurement was 430 cells/mm^3^ (IQR 290–580), measured at median 21.3 weeks gestation (IQR 15.6–26.7); 36 % (414/1165) had a CD4 count ≤350 cells/mm^3^ and 15 % (204/1321) had WHO Stage 3 or 4 HIV disease. Almost all (96 %, 1451/1509) received antenatal ART but most discontinued this at delivery, with only a quarter (410/1498) still on treatment at postnatal cohort enrolment. Only 53 % (800/1513) of women had an antenatal HIV RNA measurement; 373 women had a measurement available within 90 days (median 37 days) before delivery and while on ART, of whom 42 % (158) were virologically suppressed after a median of 9.7 weeks (IQR 5.3–13.4) on treatment.

HSV-2 seroprevalence was 68 % (1026/1513); for 87 % (1309/1509), the HSV-2 antibody test was conducted during pregnancy, for 6 % (88/1509) prior to conception and 7 % (112/1509) after delivery. Table 1 presents maternal and delivery characteristics stratified by HSV-2 serostatus.

**Table 1:** Maternal socio-demographic and clinical characteristics, stratified by HSV-2 status

Characteristic	HSV-2 seronegative *n* (%)	HSV-2 seropositive *n* (%)	*χ* ^2^ value and *p* value
Maternal age (*n* = 1512)			*χ* ^2^ = 20.34 *p* <0.001
16–23 years	116 (24)	155 (15)	
24–26 years	148 (30)	302 (29)	
27–30 years	119 (24)	319 (31)	
31 years	104 (21)	249 (24)	
Marital status (*n* = 1509)			*χ* ^2^ = 0.82 *p* = 0.364
Married/cohabiting	415 (85)	891 (87)	
Single/divorced/widowed	71 (15)	132 (13)	
Age at leaving full-time education (*n* = 1088)			*χ* ^2^ = 4.39 *p* = 0.111
≤16 years	53 (21)	222 (27)	
17–18 years	65 (26)	230 (27)	
≥19 years	133 (53)	385 (46)	
Parity (*n* = 1423)			*χ* ^2^ = 16.70 *p* <0.001
0	245 (53)	402 (42)	
1	165 (36)	392 (41)	
≥2	54 (12)	165 (17)	
Previous pregnancy terminations (*n* = 1398)			*χ* ^2^ = 63.35 *p* <0.001
0	280 (62)	373 (40)	
1	79 (17)	214 (23)	
≥2	95 (21)	357 (38)	
Pregnancy planned (*n* = 1501)			*χ* ^2^ = 3.44 *p* = 0.064
No	130 (27)	320 (31)	
Yes	355 (73)	696 (69)	
Has access to family planning (*n* = 1087)			*χ* ^2^ = 28.02 *p* <0.001
No	75 (16)	39 (6)	
Yes	388 (84)	585 (94)	
Timing of HIV diagnosis (*n* = 1513),			*χ* ^2^ = 6.06 *p* = 0.109
Before pregnancy	167 (34)	407 (40)	
First or second trimester	264 (54)	532 (52)	
Third trimester/delivery	56 (12)	87 (8)	
History of IDU (*n* = 1504)			*χ* ^2^ = 6.48 *p* = 0.011
No	409 (84)	800 (79)	
Yes	77 (16)	218 (21)	
Partner’s HIV status^a^ (*n* = 1352)			*χ* ^2^ = 36.95 *p* <0.001
Unknown	137 (32)	238 (26)	
Negative	144 (34)	210 (23)	
Positive	146 (34)	477 (52)	
HCV serostatus (*n* = 1402)			*χ* ^2^ = 46.82 *p* <0.001
Negative	344 (79)	589 (61)	
Positive	89 (21)	380 (39)	
Hepatitis B surface antigen (*n* = 1451)			*χ* ^2^ = 18.89 *p* <0.001
Negative	409 (87)	756 (77)	
Positive	62 (13)	224 (23)	
Antenatal/intrapartum antiretrovirals (*n* = 1508)			*χ* ^2^ = 31.42 *p* <0.001
cART	183 (38)	542 (53)	
ZDV +/− sdNVP	283 (58)	443 (43)	
None or sdNVP only	19 (4)	38 (4)	

^a^ As reported by the woman

### Factors associated with positive HSV-2 seropositivity

The proportion of women with HSV-2 antibodies increased significantly with age, from 57 % of women aged 16–23 years to 71 % of those aged >30 years, with higher parity and history of pregnancy termination also associated with increased probability of positive HSV-2 serostatus (Table 1). With respect to other STIs, 1.9 % (9/481) of HSV-2 seronegative women and 2.0 % (20/981) of HSV-2 seropositive women had positive serology for syphilis (Fisher’s exact test *p* = 1.0), 10.6 % (43/405) and 31 % (158/511) respectively had a positive chlamydia test (*χ*^2^ = 54.37, *p* <0.01) and 0.5 % (2/430) and 0.3 % (2/759) respectively for gonorrhoea (Fisher’s exact test *p* = 0.62). Women with HSV-2 antibodies were more likely to have a history of IDU, be positive for HCV antibodies and HBsAg, and report an HIV-positive partner (Table 1). HSV-2 seropositive women were more likely to report their most recent pregnancy as having been unplanned (Table 1, *p* = 0.06), but reported better access to family planning than HSV-2 seronegative women at cohort enrolment (Table 1).

A greater proportion of women with HSV-2 antibodies had been diagnosed with HIV before conception (Table 1) and 8 % (81/973) conceived on ART compared with 4 % (18/466) of seronegative women (*χ*^2^ = 9.79, *p* <0.01). Among those initiating ART during pregnancy, HSV-2 seropositive women were more likely to receive cART than seronegative women (51 % (451/892) vs. 37 % (165/448) respectively, *χ*^2^ = 22.64, *p* <0.01) and a slightly higher proportion had a pre-ART CD4 count of ≤350 cells/mm^3^ (38 % (249/649 vs. 32 % (56/176), *χ*^2^ = 2.55, *p* = 0.11). Of 455 women who had a viral load measure before ART start (373 (82 %) of whom were HSV-2 seropositive), median viral load was 11369 copies/ml (IQR 2392, 38676), with no difference by HSV-2 serostatus (Wilcoxon rank sum test *p* = 0.76). Overall 16 % (154/934) of HSV-2 seropositive women had WHO stage 3 or 4 disease compared with 13 % (50/387) of HSV-2 seronegative women (*χ*^2^ = 2.67, *p* = 0.10).

The multivariable model exploring factors associated with HSV-2 seropositivity excluded access to family planning, because the higher levels of access reported by HSV-2 seropositive women may have been due to treatment-seeking for symptomatic STIs (including genital ulcer disease) or termination of pregnancy and therefore may post-date HSV-2 seroconversion. Chlamydia was also excluded because, as an often transient bacterial infection, it is likely to have been acquired in the period immediately preceding testing and after chronic HSV-2 infection. In the final adjusted model, women with a history of pregnancy termination (vs no history), who reported their partner to be HIV-positive (vs of unknown HIV status) and who had HCV antibodies remained at increased risk of HSV-2 seropositivity (Table 2).

**Table 2:** Factors associated with a maternal positive HSV-2 serostatus

	Proportion of women with HSV-2 antibodies	PR (95 % CI)	*p* value	Adjusted PR (95 % CI) *n* = 1095	*p* value
Maternal age					
16–23 years	57 % (155/271)	1.00		1.00	
24–26 years	67 % (302/450)	1.19 (1.04–1.37)	0.014	1.11 (0.97–1.28)	0.137
27–30 years	73 % (319/438)	1.25 (1.09–1.44)	0.002	1.09 (0.95–1.26)	0.218
≥31 years	71 % (249/353)	1.23 (1.07–1.43)	0.004	1.03 (0.88–1.21)	0.676
Parity					
0	62 % (402/647)	1.00		1.00	
1	70 % (392/557)	1.13 (1.03–1.23)	0.009	1.01 (0.91–1.10)	0.913
≥2	75 % (165/219)	1.14 (1.01–1.28)	0.027	0.99 (0.87–1.12)	0.857
History of pregnancy termination					
0	57 % (373/653)	1.00		1.00	
1	73 % (214/293)	1.24 (1.11–1.38)	<0.001	1.22 (1.10–1.36)	<0.001
≥2	79 % (357/452)	1.36 (1.25–1.49)	<0.001	1.30 (1.18–1.43)	<0.001
Pregnancy planned					
No	71 % (320/450)	1.00		1.00	
Yes	66 % (696/1051)	0.96 (0.88–1.05)	0.343	1.03 (0.94–1.13)	
History of IDU					
No	66 % (800/1209)	1.00		1.00	
Yes	74 % (218/295)	1.05 (0.96–1.16)	0.299	0.93 (0.83–1.03)	0.174
Partner’s HIV status^a^					
Unknown	63 % (238/375)	1.00		1.00	
Negative	59 % (210/354)	0.90 (0.79–1.04)	0.147	0.91 (0.79–1.05)	0.184
Positive	77 % (477/623)	1.21 (1.10–1.33)	<0.001	1.15 (1.05–1.26)	0.003
HCV serostatus					
Negative	63 % (589/933)	1.00		1.00	
Positive	81 % (380/469)	1.25 (1.16–1.35)	<0.001	1.23 (1.13–1.35)	<0.001
Hepatitis B surface antigen					
Negative	65 % (756/1165)	1.00		1.00	
Positive	78 % (224/286)	1.20 (1.11–1.31)	<0.001	1.06 (0.97–1.16)	0.179

^a^ As reported by the woman

Although IDU history was associated with HSV-2 seropositivity among 1402 women in univariable analysis, this association was not apparent among the 1095 women included in the adjusted model (Table 2), indicating some bias in the complete-case analysis. However, among 1394 women with both IDU history and HCV serostatus available, the association between HSV-2 antibodies and HCV seropositivity after adjusting for IDU history remained (APR 1.31 95 % CI 1.21–1.42) with no association between HSV-2 antibodies and IDU after adjusting for HCV antibodies (APR 0.95 95 % CI 0.86–1.03), indicating that the observed association between HCV and HSV-2 seropositivity was not explained by factors specific to IDUs (e.g. sexual risk behaviours). Of note, 22 % (249/1119) of women without an IDU history in this population had HCV antibodies vs 79 % (217/275) of those with an IDU history.

### Infants and MTCT

Delivery and infant data are presented in Table 3. Infants of HSV-2 seropositive mothers were more likely to be delivered vaginally and to be of low birthweight than other infants (Table 3). Almost all (99.4 %, 1497/1506) infants were exclusively formula fed. At the time of analyses, all children were at least 2 years of age (median 4.98 years, IQR 3.76–6.26) and DNA PCR test results were available for 80 % (1216/1513). HIV status was available for 89 and 83 % of infants of HSV-2 seropositive and -negative women respectively (*χ*^2^ = 11.86, *p* <0.01); 37 infants were HIV-infected, giving an overall HIV MTCT rate of 2.8 % (95 % CI 1.98–3.84) (Table 3). There was no evidence that HSV-2 seropositivity was associated with HIV MTCT risk in unadjusted analyses (Tables 3 and 4, HIV MTCT rate was 3.2 % among HSV-2 seronegative women and 2.6 % among HSV-2 seropositive women, *χ*^2^ = 0.37, *p* = 0.54).

**Table 3:** Delivery and infant characteristics, stratified by maternal HSV-2 serostatus

	HSV-2 seronegative *n* (%)	HSV-2 seropositive *n* (%)	*χ* ^2^ value and *p* value
Total live births	487	1026	–
Mode of delivery (*n* = 1483)			*χ* ^2^ = 73.17 *p* <0.001
Vaginal	277 (58)	784 (78)	
Elective CS	180 (38)	176 (18)	
Emergency CS	20 (4)	46 (5)	
Gestational age (*n* = 1505)			*χ* ^2^ = 0.45 *p* = 0.501
≥37 completed weeks	445 (92)	928 (91)	
<37 completed weeks	39 (8)	93 (9)	
Birth weight (*n* = 1508)			
≥2500 g	441 (91)	890 (87)	*χ* ^2^ = 4.25 *p* = 0.039
<2500 g	45 (9)	132 (13)	
HIV infection status (*n* = 1513)			*χ* ^2^ = 12.22 *p* <0.01
Infected	13 (2.67)	24 (2.34)	
Uninfected	391 (80.29)	892 (86.94)	
Indeterminate/unknown	83 (17.04)	110 (10.72)	
MTCT rate (%) where infant	3.2 % (95 % CI 1.7–5.4)	2.6 % (95 % CI 1.7–3.9)	*χ* ^2^ = 0.37, *p* = 0.54
HIV status known (*n* = 1320)

**Table 4:** Factors associated with MTCT of HIV

	Number with MTCT (%)	Odds ratio (95 % CI)	Adjusted odds ratio (95 % CI), *p* value *n* = 1286^a^
Length of gestation			
≥37 completed weeks	33/1204 (2.7)	1	1
<37 completed weeks	4/109 (3.7)	0.73 (0.25–2.09) *p* = 0.553	0.93 (0.30–2.82) *p* = 0.891
Mode of delivery			
Vaginal	27/942 (2.9)	1	1
Elective CS	9/305 (3.0)	1.03 (0.48–2.21) *p* = 0.945	0.76 (0.32–1.80) *p* = 0.529
Emergency CS	1/58 (1.7)	0.58 (0.08–4.38) *p* = 0.602	1.04 (0.13–8.28) *p* = 0.973
Antenatal/intrapartum ARVs			
cART	11/607 (1.8)	1	1
ZDV +/− sdNVP	20/666 (3.0)	0.61 (0.29–1.28) *p* = 0.190	0.59 (0.27–1.31) *p* = 0.194
None or sdNVP only	6/42 (14.3)	5.31 (2.01–14.03) *p* <0.01	3.42 (1.19–9.85) *p* = 0.023
IDU history			
No	30/1057 (2.8)	1	1
Yes	7/254 (2.8)	0.98 (0.43–2.26) *p* = 0.964	1.18 (0.46–2.99) *p* = 0.735
HSV-2 serostatus			
Negative	13/404 (3.2)	1	1
Positive	24/916 (2.6)	0.80 (0.41–1.60) *p* = 0.553	1.43 (0.54–3.77) *p* = 0.474

^a^ Adjusted additionally for time period of delivery (2007–09 vs. 2010–12) and including random effect term for centre of enrolment

In multivariable analyses, the only factor associated with HIV MTCT was lack of antenatal ART, associated with a three-fold increased risk (Table 4). In the first of the three sub-analyses of factors associated with HIV MTCT risk, adjusting additionally for maternal syphilis serology among 1241 mother-child pairs with this available, the AOR for positive versus negative HSV-2 serostatus was 1.39 (95 % CI 0.51–3.80, *p* = 0.523). In the sub-analysis restricted to the 927 women delivering vaginally the AOR for HSV-2 seropositive vs seronegative was 1.46 (95 % CI 0.40–5.34 *p* = 0.565) and in the sub-analysis restricted to 629 women without antenatal cART, the AOR was also not significantly increased (AOR 1.57, 95 % CI 0.30–8.12, *p* = 0.590).

## Discussion

In this large cohort of HIV-positive pregnant women, we found that two-thirds had antibodies to HSV-2. Almost all women received antenatal ART and around half received cART; the HIV MTCT rate was 2.8 % overall, with no increased risk of MTCT of HIV detected among HSV-2 seropositive women.

Recent global estimates of prevalent HSV-2 in adults aged 15–49 years in 2012 indicated prevalence in females of 14.8 % and in males of 8.0 %; in Europe the estimate was 10 % for women and 4 % for men [20]. High HSV-2 prevalence is reported in women living with HIV, for example, 86 % of HIV-positive pregnant women in a Nairobi study [21]. In a Canadian study of Africa/Caribbean-born women, prevalence of HSV-2 infection was 86 % in HIV-positive and 47 % in HIV-negative women [22]. Thus our finding of a seroprevalence of 68 % is consistent with published estimates.

HSV-2 is a persistent infection and thus prevalence increases with age, as seen here and demonstrated elsewhere. In the global estimates study, prevalence increased from <5 % in 15–19 year olds, to 13 % by age 30–34 and 17 % by age 45–49 [20]. In our adjusted analyses we found that HSV-2 seropositive women were more likely to have a history of pregnancy termination, which may be a marker of greater sexual risk, including lack of or inconsistent use of barrier contraception. In this population, condoms are the main form of contraception [23] which is an ineffective family planning method and of only moderate effectiveness in preventing acquisition of HSV-2 [24]. Other factors associated with significantly greater probability of HSV-2 seropositivity were having a known HIV-positive partner and being co-infected with HCV. The epidemiology of HCV infection in Ukraine is not well understood with over a fifth of non-IDUs in this cohort reported to be HCV seropositive. HCV is more sexually transmissible among HIV-positive individuals [25] and our findings of an association between HCV and HSV-2 seropositivity may indicate shared risk factors for sexual acquisition. However, risk factors for HCV acquisition in this setting may also include iatrogenic exposures, which could have been more common among HSV-2 seropositive women given their greater likelihood of history of pregnancy termination.

We did not detect an association between HSV-2 antibodies and increased risk of HIV MTCT; this is in contrast with findings from other studies, which reported an association between HSV-2 antibodies or genital ulcer disease and HIV MTCT [15, 16, 26, 27]. These studies were all conducted more than 10 years ago in populations with much higher background rates of HIV MTCT. Our sample size of 404 HSV-2 seronegative and 916 seropositive mothers and 3.2 % transmission rate in the seronegative group allowed us to rule out a 2.25-fold increased risk of HIV MTCT with HSV-2 antibodies (i.e. a transmission rate of ≥7.2 %), with 80 % power and alpha 0.05. To have detected a 50 % increased risk, as previously reported in the Zimbabwean study [27], would have required a much larger sample size of around 1850 HSV-2 seronegative and 4200 HSV-2 seropositive women, given the low HIV MTCT rate overall. Therefore, although we did not detect an association between HSV-2 antibodies and increased risk of HIV MTCT, our study did not have sufficient statistical power to rule out the smaller increases in HIV MTCT risk associated with HSV-2 seropositivity found previously, due to the low HIV MTCT rates overall in our study population, almost all of whom received antenatal ART.

Although we showed a significant association between MTCT and use of ART, we were unable to explore the association between HSV-2 serostatus and MTCT after adjusting for maternal HIV viral load because of limited viral load testing: of the 37 HIV-infected infants, 23 were born to women with no antenatal HIV RNA measure available and only five mothers of the remaining 14 had a measurement available in the 90 days preceding delivery. The HIV MTCT rate reported here was substantially lower than that observed in the Ukraine ECS overall in the same time period (4.56 %, 95 % CI 4.04–5.13), because our analysis was restricted to a postnatal cohort who generally had better access to PMTCT interventions, reflecting greater engagement with antenatal and HIV care services [13].

The high prevalence of HSV-2 found in our HIV-positive pregnant women has implications for their infants. Neonatal HSV-2 infection is a serious condition associated with high morbidity and mortality, with antiviral prophylaxis for HSV-2 recommended during pregnancy in women with frequent clinical recurrences. In HIV co-infected women this could have the additional benefit of reducing HIV viral load and/or HIV MTCT risk. However, risk of vertical transmission of HSV-2 is higher in primary infection during pregnancy (around 40 %) compared to recurrent infections (1–3 %) [28]. PCR test results and data on clinical symptoms were not available, and we were therefore unable to make conclusions about the timing of HSV-2 seroconversions here or prevalence of genital ulcer disease during pregnancy, however most HSV-2 seropositive women had been tested during pregnancy (87 %) or pre-conception (6 %). Of HSV-2 seronegative women, 1 in 10 had a recent chlamydia diagnosis, indicating ongoing sexual risk and potential risk of HSV-2 acquisition during pregnancy. Up to 20 % of HSV-2 seronegative women with an HSV-2-infected partner will seroconvert during pregnancy, with highest risk of neonatal infection if this occurs late in pregnancy [29]. Some experts suggest partner testing of pregnant women susceptible to HSV-2 and counselling of those with a positive partner about abstinence and reducing oral-genital contact near term [30].

As the postnatal cohort enrols women retained in HIV care following delivery, our study population may have had better uptake of sexual and reproductive health services. Our results may therefore not be generalizable to the entire HIV-positive childbearing population in Ukraine; in reality, HSV-2 seroprevalence may be higher if women not engaged with services after delivery were also more likely to have HSV-2 co-infection. More research is needed on occurrence of genital ulcer disease among HSV-2 seropositive women and outcomes of their neonates, and more generally about sexual health of women in Ukraine living with or at high risk of acquiring HIV, to strengthen preventative health services

## Conclusion

In conclusion, we found a high prevalence of HSV-2 antibodies but no associated increased risk of vertical HIV transmission, in this population with an overall HIV MTCT rate of 2.8 %. High prevalence of recent chlamydia diagnosis indicated ongoing sexual risk, suggesting that HSV-2 seronegative women may benefit from counselling on prevention of HSV-2 acquisition during pregnancy and that clinicians should be alert to the possibility of a primary maternal infection, while HSV-2 seropositive women with clinical symptoms require appropriate obstetric management.

## Declarations

### Ethics approval and consent to participate

The Women’s Study is nested within the ECS, which has ethical approval from the Great Ormond Street Hospital for Children NHS Trust/Institute of Child Health Research Ethics Committee (reference 96EB02). Local approvals were also obtained from all the participating HIV/AIDS Centres. Women were required to give verbal consent to take part in the ECS and to complete an anonymised questionnaire at enrolment into the Women’s Study; the return of this questionnaire was taken as documentation and evidence of consent to participate in the Women’s Study.

### Consent for publication

Not applicable.

### Availability of data and materials

As our dataset contains potentially patient-identifiable data and data items on sensitive health information such as HIV infection status, other sexually transmitted infections and substance use, our data are not automatically available for sharing. We provide pseudonymised data sets for inclusion in pooled analyses and systematic reviews. We invite people who are interested in accessing our data to contact Claire Thorne for further information.

## Abbreviations

HSV 2: Herpes simplex virus type 2
HIV: human immunodeficiency virus
MTCT: mother to child transmission
STI: sexually transmitted infection
ECS: European Collaborative Study
PMTCT: prevention of MTCT
VL: viral load
IDU: injecting drug use
cART: combined antiretroviral therapy
PCR: polymerase chain reaction
DNA: deoxyribonucleic acid
RNA: ribonucleic acid
HCV: hepatitis C virus
PR: prevalence ratio
aPR: adjusted prevalence ratio
CI: confidence Interval
IQR: interquartile range
HbsAg: hepatitis B surface antigen
